# Jejunal Diverticular Hemorrhage: A Diagnostic Blind Spot in Small Bowel Bleeding

**DOI:** 10.7759/cureus.98585

**Published:** 2025-12-06

**Authors:** Nika Bakshi, Claire Wang, Samiksha Pandey, Jimmi Mangla, Michael Duffy

**Affiliations:** 1 Medicine, Oakland University William Beaumont School of Medicine, Royal Oak, USA; 2 Gastroenterology, Corewell Health William Beaumont University Hospital, Royal Oak, USA; 3 General Surgery, Corewell Health William Beaumont University Hospital, Royal Oak, USA; 4 Gastroenterology and Hepatology, Corewell Health William Beaumont University Hospital, Royal Oak, USA

**Keywords:** general surgery, gi bleeding, jejunal diverticula, small bowel gi bleeding, small bowel hemorrhage

## Abstract

Small bowel bleeding is an uncommon but important cause of gastrointestinal (GI) hemorrhage. Here, we present a case of an 82-year-old woman who presented to the emergency department with lightheadedness and diarrhea. Upon further evaluation, a small-intestine bleed was suspected. Despite multiple studies, including two computed tomography angiograms (CTA) and subsequent mesenteric arteriography, localizing the hemorrhage was challenging. Due to ongoing clinical deterioration, a decision was made to pursue surgical exploration, which ultimately resulted in resection of the bleeding jejunal diverticulum.

## Introduction

Jejunal diverticula are rare outpouchings of the small intestine with an incidence between 0.06% and 1.5% [1]. Notably, this incidence is likely higher since the majority of jejunal diverticula are asymptomatic, reported in up to 80% of cases [1,2]. If symptomatic, patients often experience symptoms such as bloating and epigastric pain [1]. They can also lead to serious complications such as gastrointestinal (GI) bleeding, perforation, and obstruction in 18% of cases [1-3]. Patients with gastrointestinal bleeding often do not display previous GI symptoms and often develop acute-onset hemorrhage, making early detection and timely intervention imperative [1].

Diagnosing bleeding from the small intestine, especially the distal jejunum, is particularly challenging due to limited endoscopic access, intermittent bleeding, and the length of the bowel [4]. For example, in patients with gastrointestinal bleeding, approximately 5%-10% will remain undetected on standard endoscopic evaluation [4]. Modern imaging, such as computed tomography angiography (CTA) and CT enterography (CTE), can also be limited due to intermittent bleeding patterns and hemodynamic instability, or in patients with severe kidney impairment [4].

In this case report, we present a case of a massive jejunal diverticular bleed in an elderly patient whose bleeding remained undetected despite multimodal evaluation. This case highlights the diagnostic challenges of small bowel bleeding, and in particular, jejunal diverticular bleeding, and emphasizes the importance of urgent laparotomy in these cases.

## Case presentation

**Figure 1:**
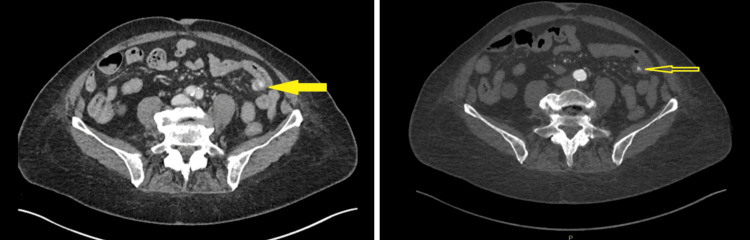
Sagittal CTA images showing active jejunal diverticular bleeding (yellow arrows) (A) Initial sagittal slice showing contrast extravasation in the jejunum. (B) Adjacent sagittal slice highlighting the same bleed at a slightly different level. CTA: computed tomography angiogram

An 82-year-old woman with a past medical history of type 2 diabetes mellitus, hypertension, and atrial fibrillation, not on anticoagulation, and with no prior gastrointestinal history, presented to the emergency department with lightheadedness and diarrhea. On arrival, the patient was hemodynamically stable and afebrile. Her laboratory results were notable for iron deficiency anemia with a hemoglobin of 8.5 g/dL (reference range: 12.1-15.0 g/dL) and hematocrit of 27.5% (reference range: 35.4%-44.2%). Upon further examination, dark maroon stool was noted on the rectal examination with a positive guaiac test. Following initial resuscitation and transfusion of one unit of packed red blood cells, a computed tomography angiogram (CTA) of the abdomen and pelvis was ordered, which demonstrated no active bleed. An esophagogastroduodenoscopy (EGD) and colonoscopy were subsequently planned but were soon canceled following persistent hematochezia leading to hemodynamic instability. An emergent repeat CTA was performed, which revealed active contrast extravasation from a presumed jejunal diverticulum (Figure 1).

She underwent selective mesenteric arteriography but failed to demonstrate active bleeding. However, empirical embolization was performed at the jejunal branch correlating with the CTA findings. Following the embolization, the patient remained hemodynamically stable through the immediate postoperative period. There was no evidence of abdominal pain or further bleeding. However, despite the absence of overt bleeding, she developed recurrent hemodynamic instability with a continued decline in hemoglobin, raising concern for ongoing occult hemorrhage.

**Figure 2:**
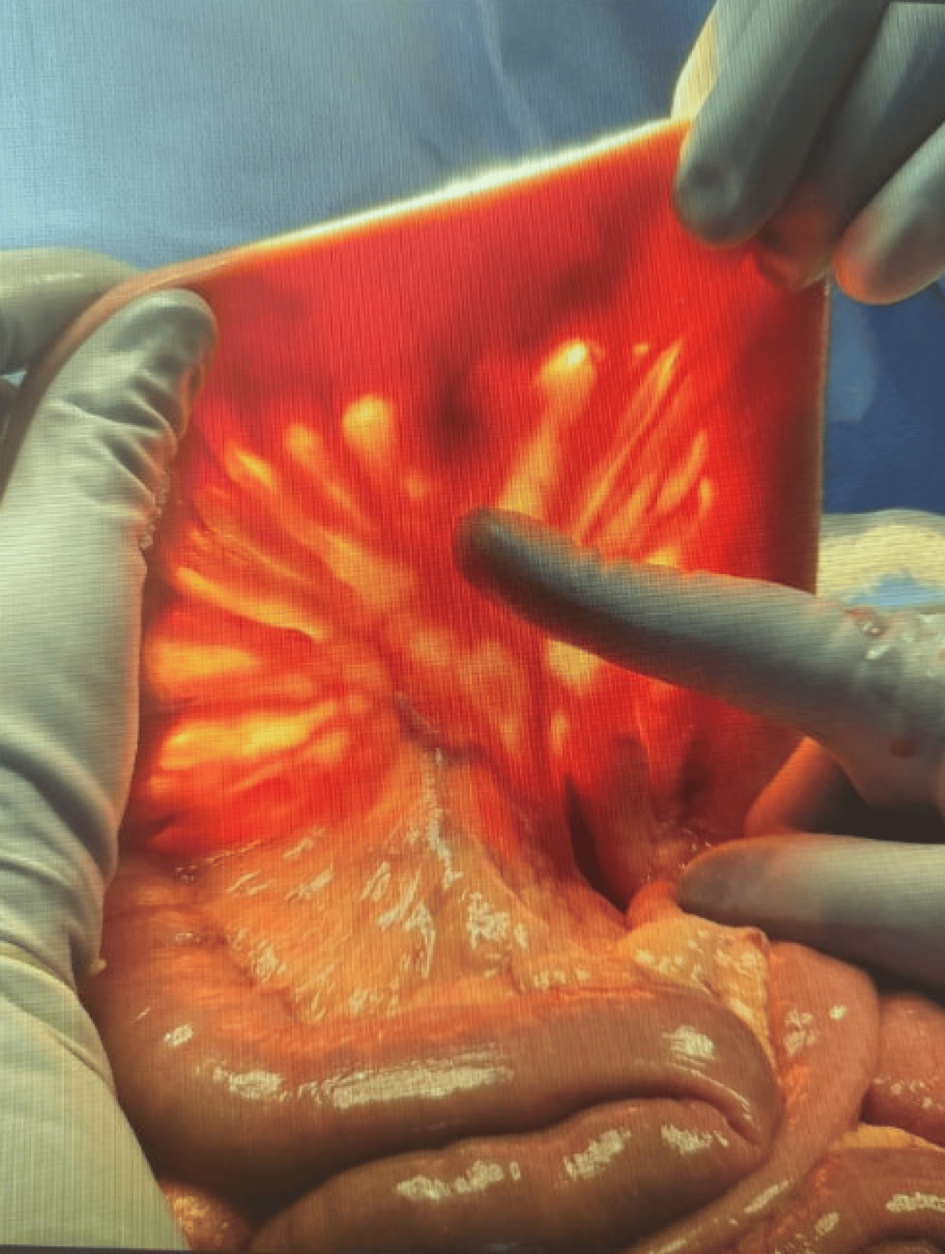
Intraoperative image showing a jejunal diverticulum on the antimesenteric border of the small intestine

Despite embolization and an initial period of hemodynamic stability, an exploratory laparotomy was pursued due to ongoing clinical deterioration. Although the bowel appeared grossly normal, transillumination revealed a bleeding jejunal diverticulum on the mesenteric side (Figure 2). Segmental small bowel resection was performed, and histopathologic evaluation confirmed a benign jejunal diverticulum.

## Discussion

Jejunal diverticula are a rare cause of small bowel bleeding, with a reported prevalence of 1.1%-2.3% [5]. It typically affects elderly patients over the age of 60, but cases have been reported in younger adults [6-8]. Although primarily asymptomatic, GI bleeding occurs in approximately 3.4%-8.1% of patients, with bleeding often being massive and recurrent [5]. Complications such as perforation and obstruction can also occur, and up to 10%-30% of patients require surgical intervention [3,9]. Despite its rarity, the challenge in these cases is not due to the etiology but rather the difficulty of acutely localizing the bleeding source.

The small intestine is inherently difficult to evaluate due to its length and limited accessibility with conventional endoscopic modalities. One of the standard diagnostic approaches to small bowel bleeding in hemodynamically stable patients is esophagogastroduodenoscopy (EGD) [4]. However, EGD can often fail to identify the source when bleeding originates distal to its reach, particularly in the distal jejunum. In hemodynamically unstable patients, such as ours, a CTA is a part of the initial evaluation since it can rapidly localize active extravasation [10]. However, the diagnostic performance of CTA is highly dependent on the bleeding rate [10]. According to Murphy et al., a CTA can typically detect a bleeding rate of 0.5-1 mL/minute, which is equivalent to a loss of approximately 2-3 units of blood over 24 hours [10]. Moreover, a meta-analysis of nine studies including 198 patients with overt GI bleeding demonstrated a sensitivity of 89% and specificity of 85% for CTA in identifying active GI bleeding [10]. Despite this high degree of diagnostic accuracy, a CTA can still fail to detect a hemorrhage when GI bleeding is intermittent, as in our patient [10].

While CTA is the study of choice for hemodynamically unstable patients with overt bleeding, CTE is the more appropriate choice in hemodynamically stable patients with obscure or occult bleeding [4]. A meta-analysis of 18 studies reported a pooled diagnostic yield of 40% for CTE [4]. This low diagnostic yield underscores the limitations of CTE and its challenges in localizing small bowel bleeding sources.

In stable patients, capsule endoscopy is often the preferred next diagnostic test following a negative EGD and colonoscopy [4]. Capsule endoscopy offers distinct advantages, including noninvasive evaluation of the entire small bowel [4]. In the setting of ongoing overt bleeding, its diagnostic yield exceeds 90% [4]. However, similar to CTA, capsule endoscopy may fail to identify the bleeding source when hemorrhage is intermittent, further complicating timely diagnosis [4].

Furthermore, enteroscopy, including single-balloon, double-balloon, and spiral techniques, can be used for both diagnostic and therapeutic purposes [2]. However, these modalities are resource-intensive, require specialized expertise, and are often unavailable in many centers [2]. In addition, they are not feasible in hemodynamically unstable patients such as ours. Push enteroscopy is also useful for evaluating small bowel bleeding; however, its reach is limited and might miss distal jejunal lesions [4].

Our patient's clinical course highlights many of these diagnostic limitations. Similar to what is documented in literature, her bleeding being intermittent made it difficult to localize on CTA. EGD and colonoscopy also could not be performed due to hemodynamic instability, further limiting the range of diagnostic studies mentioned. Although repeated CTA demonstrated jejunal extravasation, these findings were not definitive. Definitive diagnosis was ultimately made during surgical exploration, which allowed for resection of the bleeding diverticulum. This demonstrates that despite the range of diagnostic studies outlined above, localizing small bowel bleeding remains challenging, especially when the hemorrhage is intermittent. In cases of ongoing clinical deterioration or when noninvasive testing is inconclusive, early consideration of surgical exploration is warranted.

## Conclusions

This case illustrates the difficulty in identifying small bowel bleeding, especially when the hemorrhage is intermittent and imaging results are inconclusive. While CTA for an upper GI bleed has a sensitivity of 50%-70%, it is insufficient for detecting intermittent bleeding. In our case, ongoing clinical deterioration necessitated surgical exploration, which ultimately established the diagnosis. This case underscores the importance of timely decision-making and an interdisciplinary approach to managing complex gastrointestinal bleeding. When noninvasive imaging fails to localize the source and the patient continues to decompensate, early surgical intervention should be strongly considered. The coordinated efforts of gastroenterology, interventional radiology, and surgery were essential to achieving definitive management in this case.

## References

[1] Lee BJ, Kumar P, Van den Bosch R (2015). Jejunal diverticula: a rare cause of life-threatening gastrointestinal bleeding. J Surg Case Rep.

[2] Chiorescu S, Mocan M, Santa ME, Mihăileanu F, Chiorescu RM (2024). Acute complicated jejunum diverticulitis: a case report with a short literature review. Front Med (Lausanne).

[3] Giannopoulos P, Linardoutsos D, Tsamis D, Spirakopoulos P, Sklika E, Patrinios G (2013). Perforated jejunal diverticulosis: report of a case and review of the literature. J Med Cases.

[4] Cave D (2025). Evaluation of suspected small bowel bleeding (formerly obscure gastrointestinal bleeding). UpToDate.

[5] Gunjan D, Sharma V, Rana SS, Bhasin DK (2014). Small bowel bleeding: a comprehensive review. Gastroenterol Rep (Oxf).

[6] Isthiyak AR, Ariyaratne HL, Sallay NS, Thariq T (2025). Jejunal diverticulum forming an Interloop abscess in a young adult: a rare case report. Glob J Surg Case Rep.

[7] Jawed A, Jawed A, Kumari S, Shaikh OA, Nashwan AJ (2023). A rare case of perforated jejunal diverticula of an uncommon origin. Clin Case Rep.

[8] Alam S, Rana A, Pervez R (2014). Jejunal diverticulitis: imaging to management. Ann Saudi Med.

[9] Butler JS, Collins CG, McEntee GP (2010). Perforated jejunal diverticula: a case report. J Med Case Rep.

[10] Murphy B, Winter DC, Kavanagh DO (2019). Small bowel gastrointestinal bleeding diagnosis and management-a narrative review. Front Surg.

